# Hot or cold? Feasibility, safety, and outcome after radiofrequency‐guided versus cryoballoon‐guided left atrial appendage isolation

**DOI:** 10.1002/joa3.12691

**Published:** 2022-03-08

**Authors:** Shota Tohoku, Shaojie Chen, Stefano Bordignon, Julian Kyoung‐Ryul Chun, Boris Schmidt

**Affiliations:** ^1^ Cardioangiologisches Centrum Bethanien (CCB), Frankfurt Academy For Arrhythmias (FAFA); Abteilung für Kardiologie, Medizinische Klinik III Agaplesion Markus Krankenhaus Frankfurt am Main Germany; ^2^ Die Sektion Medizin Universität zu Lübeck Lübeck Germany; ^3^ Universitätsklinikum Frankfurt Medizinische Klinik 3‐ Klinik für Kardiologie Frankfurt Germany

**Keywords:** ablation, atrial fibrillation, cryoballoon, left atrial appendage isolation

## Abstract

**Background:**

Left atrial appendage (LAA) isolation (LAAI) has been described as an adjunctive ablation strategy for patients with recurrent atrial tachyarrhythmia (ATa).

**Objectives:**

We compared the clinical impact of persistent durable LAAI between radiofrequency (RF)‐guided wide‐area LAAI and cryoballoon (CB)‐guided ostial LAAI.

**Methods:**

Consecutive patients who underwent RF‐ or CB‐guided LAAI were retrospectively analyzed. RF‐guided LAAI was performed by combining linear ablation. CB‐guided LAAI was performed by LAA ostial ablation. Following LAAI, the patients underwent an invasive remapping study. LAA closure was conducted if persistent durability was confirmed. The procedural data, LAAI durability, and ATa recurrence were assessed.

**Results:**

A total of 260 patients (RF: *n* = 201; CB: *n* = 59) undergoing LAAI were identified. The acute rate of procedural LAAI was higher in the CB group (CB:94.9% vs. RF:82.6%, *p* = .02) with a lower pericardial effusion incidence (CB:0% vs. RF:7.5%, *p* = .03). The 6‐week durable LAAI was similar between the two groups (RF:78.3% vs. CB:66.0%, *p* = .103). During follow‐up, one gastrointestinal bleeding and four stroke events including one subsequent intracranial bleeding leading to death occurred in the RF group, while one gastrointestinal bleeding occurred in the CB group.

The 1‐year ATa recurrence‐free rate was higher in patients with durable LAAI following RF‐guided LAAI (RF:76.3% vs. CB:56.7%, *p* = .0017). Multivariate analysis revealed RF‐guided LAAI as a predictor of freedom from ATa recurrence (HR: 0.478, 95%CI: 0.336–0.823, *p* = .017).

**Conclusions:**

LAAI can be more readily and safely achieved by CB‐guided ostial ablation. In patients with confirmed LAAI, however, the freedom from ATa recurrence was higher after RF‐guided wide‐area isolation.

## Graphical abstract

Durable LAAI: Wide‐Area RF ablation vs. Ostial CB ablation.

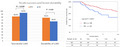

## 1 INTRODUCTION

Catheter ablation is an established therapeutic option for symptomatic patients with atrial fibrillation (AF).^1^, ^2^ Furthermore, pulmonary vein (PV) isolation (PVI) remains the procedural cornerstone in catheter ablation of AF.^1^ However, despite durable PVI,^3^ patients still suffer from AF recurrence, thus suggesting that non‐PV triggers play a significant role in initiating and maintaining AF. Left atrial appendage (LAA) has been recognized as ectopic firing foci for AF development, and the electrical LAA isolation (LAAI) on top of PVI can significantly improve clinical outcomes among patients with persistent AF.^4^

Different techniques for LAAI, such as radiofrequency (RF)‐guided wide‐area LAAI^5^ and cryoballoon (CB)‐guided ostial LAAI,^6^ have been previously described. Although these two ablation approaches have the same electrophysiological endpoint, the ablation concept differs with respect to the amount of atrial tissue enclosed into the lesion set. While empirical LAAI has been reported to improve the clinical results of AF ablation,^4^ the rate of persistent LAAI has been questioned by various remapping studies.^5^, ^6^, ^7^

This study compared the procedural profile and clinical outcomes of LAAI using two ablation strategies.

## 2 METHODS

### 2.1 Study population

This is a retrospective observational study. Patients with symptomatic, drug‐refractory AF who underwent LAAI were analyzed between July 2011 and February 2020. Of those, patients whose PV was persistently isolated were enrolled in the follow‐up analysis. Conversely, patients who underwent surgical ablation or previous left atrial appendage closure (LAAC) were excluded.

All patients provided written informed consent before undergoing the ablation procedure. The study was approved by the Institutional Review Board and complied with the Declaration of Helsinki.

### 2.2 Indication of LAAI

In principle, LAAI was considered for patients with symptomatic AF, despite durable PVI, and/or atrial tachyarrhythmias (ATa) involving the LAA or AF emanating from the LAA (AF initiated by LAA triggers after intraprocedural electrical cardioversion, or atrial tachycardia including high rate of atrial premature complex emanating from the LAA during sinus rhythm). RF‐based LAAI was preferred in patients with a history of atrial tachycardia. Along the time course, the indication of CB‐guided LAAI was further expanded for patients who were prescribed antiplatelet therapy on the basis of the initial experience reports.^5^, ^6^

### 2.3 Electrophysiological procedure

The procedural methods have been already described in previous research studies.^5^, ^6^, ^8^ All patients were under deep sedation. After positioning a 6F‐decapolar catheter into the coronary sinus, double transseptal punctures for RF‐guided LAAI or a single transseptal puncture for CB‐guided LAAI was performed using a modified Brockenbrough technique. Intravenous heparin was administered targeting an activated clotting time of 300–350 s. Selective PV and LAA angiography were performed under standard right anterior oblique (RAO 30°) and left anterior oblique (LAO 40°).

### 2.4 Catheter ablation protocol

#### 2.4.1 LAAI with RF

**FIGURE 1:**
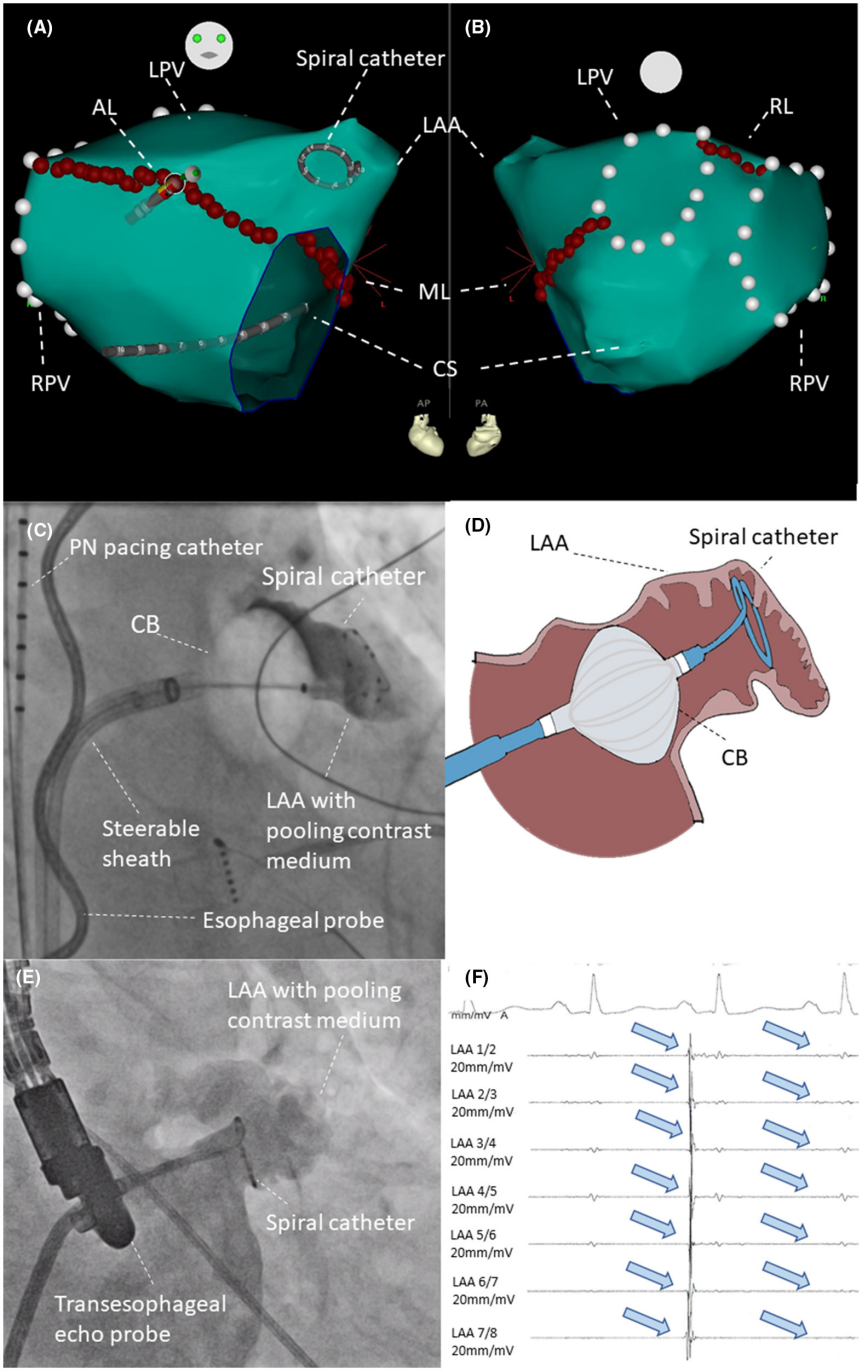
(A, B) Three‐dimensional map of the left atrium using CARTO 3 with radiofrequency current (RF)‐guided MAZE‐like left atrial appendage isolation (LAAI) in anteroposterior (A) and posteroanterior (B) views. AL, Anterior block line; CS, Coronary sinus; LAA, Left atrial appendage; LPV, Left‐sided pulmonary veins; ML, Mitral isthmus block line; RL, Roof line block line; RPV, Right sided pulmonary veins. (C) Fluoroscopic image of cryoballoon(CB)‐guided LAAI in right anterior oblique (RAO) 30°. (D) The schema of Figure 1C. (E) Fluoroscopic image of re‐mapping study using spiral mapping catheter in RAO 30°. (F) Intracardiac electrogram at the re‐mapping study. Blue allows means dissociated isolated electrical potential of LAA

The ablation method has been previously described.^5^ PVs were remapped using a spiral‐mapping catheter to confirm durable PVI. Following LAA angiography, a spiral‐mapping catheter was advanced into the LAA to record the LAA electrical activity. Consequently, three linear lesions in the left atrium (LA) were deployed with the goal of LAAI (Figure 1A,B): (1) a roof line (RL, with 30 W) between the superior PVs; (2) an anterior septal line (AL, with 35 W) between the mitral annulus anterior and the right superior PV; and (3) a mitral isthmus line (MIL, with 30 W) between the lateral mitral annulus and the left inferior PV. Conduction block of the linear lesions was performed via differential pacing. In case of residual conduction across the mitral isthmus line after endocardial ablation, epicardial ablation within the CS was performed. Conduction block of the three lines resulted in electrical isolation of the LAA.

#### 2.4.2 LAAI with CB

Similarly, the CB ablation technique has been described previously.^6^, ^9^ The circular mapping catheter (Achieve) was used to navigate the CB (Arctic Front Advance/Pro CB2/CB3, Medtronic) to the LAA ostium (Figure 1C,D). After inflation, LAA occlusion was assessed by contrast medium injection through the distal CB lumen. During CB‐guided LAA ablation, a 6‐Fr octapolar catheter was positioned at the left subclavian vein to stimulate the phrenic nerve (PN; 12 mV, 2.9 ms pulse‐width, cycle length of 1200 ms) and to monitor the PN function. The freeze duration was set to 240 s, and an empiric bonus freeze was delivered after electrical isolation of the LAA.^6^, ^9^ Successful LAA isolation was defined as the elimination or dissociation of the LAA potential as recorded by the spiral‐mapping catheter.

### 2.5 Post‐ablation management

A routine transthoracic echocardiogram was used to exclude pericardial effusion. According to the institutional standards, patients were discharged 2 days after the procedure in the absence of any complications. All patients were scheduled for LAAC at 6 weeks post index ablation. Oral anticoagulation (OAC) was resumed 6 h after the procedure. The anti‐arrhythmic drug treatment was routinely discontinued after the procedure.

### 2.6 Remapping study and follow‐up

We gained single venous access to perform a transseptal puncture. A spiral‐mapping catheter was used to document LAAI (Figure 1E,F). If persistent electrical conduction block of the LAA was confirmed, LAAC was then directly performed. In case of resumed LA to LAA conduction, repeat ablation with the goal of re‐LAAI was performed.

The patients were evaluated at the outpatient clinic at 3, 6, and 12 months following the last procedure. Follow‐up transesophageal echocardiogram was performed at 6 weeks and 6 months after LAAC. Finally, the patients were scheduled to undergo 72‐h Holter monitoring to record ATa recurrence during clinical follow‐up.

### 2.7 Outcome measures

Acute procedural success was defined as electrical isolation of LAA. The first 3 months after the last ablation were considered as the blanking period.

ATa recurrence was defined as any documented ATa that was sustained for more than 30 s after the last LAAI procedure.

Safety outcomes, such as procedural complications, bleeding, thromboembolic events, and death during follow‐up, were also recorded.

### 2.8 Statistical analysis

Data are expressed as mean ± SD. Categorical variables are expressed as number and percentage. For comparison between the two groups, unpaired Student's *t*‐test or *𝜒*2 test/Fisher's exact test was employed. We used logistic regression to estimate the propensity score, including the following covariates: age, gender, body mass index (BMI), history of coronary artery disease, heart failure, and number of previous procedures. Based on their propensity score, patients who underwent RF‐ or CB‐guided LAAI were matched on a 1:1 basis with the nearest neighbor algorithm, without replacement, using a caliper width 0.2 logit of the standard deviation. Logistic regression analysis was conducted to investigate the predictor for durable LAAI. The Kaplan–Meier method was used to estimate the cumulative incidence and assess potential differences using the log‐rank test. To identify independent predictors of freedom from ATa recurrence, Cox proportional‐hazard regression analysis was conducted in multivariate models. The selection of a priori variables was based on previous literature and clinical importance. Age, gender, and RF‐guided LAAI were included in all our analyses. Other clinically relevant variables were included to increase the *p*‐value according to the event number. The final multivariate model was developed for each potential confounder. A *p*‐value of <.05 was considered statistically significant. Analyses were conducted using the JMP software version 11.0 (SAS Institute). The data that support the findings of this study are not publicly available because of privacy or ethical restrictions but are available upon request from the corresponding author.

## 3 RESULTS

### 3.1 Patient population

A total of 260 patients (RF group: *n* = 201 vs. CB group: *n* = 59) underwent an index LAAI procedure. LAAI was conducted mostly in repeat procedures (RF: 90.6% vs. CB: 89.8%, *p* = .870). In the patients whose LAA demonstrated a clear involvement in ATa occurrence, LAAI was conducted exceptionally in addition to PVI or re‐PVI (RF: 11.4% vs. CB 10.2%. *p* = .785). The demographic details and procedural data are presented in Tables 1 and 2.

**TABLE 1:** Patient characteristics

	Entire cohort			Patients with persistent durable LAAI			Propensity score matched		
Patient characteristics	RF, *N* = 201	CB, *N* = 59	*p*	RF, *N* = 99	CB, *N* = 41	*p*	RF, *N* = 31	CB, *N* = 31	*p*
Age, years	71 ± 8	69 ± 10	.102	71 ± 8	69 ± 10	.338	70 ± 10	70 ± 8	.978
BMI, kg/m^2^	26.7 ± 4.2	30.4 ± 6.0	<.001	27.1 ± 4.0	29.8 ± 6.1	.003	29.1 ± 4.0	28.8 ± 5.3	.809
Male gender, %	42.8	67.8	.001	44.4	70.7	.005	61.3	67.7	.596
Non‐paroxysmal AF, %	87.6	88.1	.906	84.8	87.8	.649	74.2	83.8	.282
LA diameter, mm	43.6 ± 5.5	43.6 ± 5.9	.955	43.7 ± 5.3	43.5 ± 6.5	.836	44.6 ± 6.4	42.6 ± 6.3	.227
LVEF, *n*	58.2 ± 8.6	59.8 ± 6.8	.189	59.1 ± 7.4	59.2 ± 6.2	.940	59.3 ± 6.8	59.7 ± 7.2	.839
Hypertension, %	78.0	81.4	.580	80.8	80.5	.760	74.2	80.7	.544
Diabetes mellitus, %	9.0	8.5	1.000	10.1	7.3	.756	12.9	9.7	.688
History of stroke, %	8.5	8.5	1.000	9.1	4.9	.508	3.2	6.5	.554
Heart failure, %	29.7	20.3	.160	33.3	19.5	.152	22.6	19.4	.755
Coronary artery disease, %	16.9	28.8	.043	16.2	26.8	.145	29.0	29.0	1.000
Antiarrhythmic drug, %	30.9	27.1	.583	24.2	29.3	.536	25.8	25.8	1.000
Class IAAD, %	13.9	3.4	.034	9.1	4.9	.508	9.7	3.2	.612
Class III AAD, %	16.9	23.7	.236	15.2	24.4	.194	16.1	22.6	.520
Beta blocker, %	69.7	71.2	.821	71.7	65.9	.491	79.4	67.7	.272
Repeat procedure, %	90.6	89.8	.870	92.9	90.2	.591	100.0	87.1	.113
Number of prior ablations, n	2.4 ± 0.8	2.7 ± 1.0	.010	2.4 ± 0.8	2.8 ± 1.0	.017	2.5 ± 0.7	2.4 ± 0.8	0.521
History of linear ablation, %	37.3	40.7	.633	41.4	46.3	.592	45.2	41.9	.798
Anterior line ablation, %	31.8	35.6	.589	40.4	41.5	.908	41.9	35.5	.602
Roof line ablation, %	23.9	33.9	.124	26.3	39.0	.134	25.8	35.5	.409
Mitral isthmus ablation, %	1.5	0	1.000	1.0	0.0	1.000	3.2	0.0	1.000
History of CTI ablation, %	16.9	18.6	.758	18.2	24.4	.403	16.1	22.6	.520

Abbreviations: AF, atrial fibrillation; AAD, anti arrhythmic drug; BMI, body mass index; LA, left atrium; CTI, Cavotricuspid isthmus; LVEF, left ventricular ejection fraction.

**TABLE 2:** Procedural characteristics

Procedural characteristics	RF, *N* = 201	CB, *N* = 59	*p*
Total procedural time, min	107 ± 42	63 ± 29	<.0001
Fluoroscopic time, min	11.8 ± 6.2	8.5 ± 5.9	.0004
PVI at identical procedure, %	11.4	10.2	.785
Procedural characteristics of RFC‐guided LAAI			
Anterior line ablation, %	93.5		
Roof line ablation, %	89.5		
Mitral isthmus line ablation, %	99		
Epicardial ablation from CS distal, %	13.9		
CTI ablation, %	52.7		
Procedural characteristics of CB‐guided LAAI			
Occlusion grade (4/3), %		83/17	
LAAI at first application		79.6	
Time to sustained isolation, s		119 ± 58	
Temperature at isolation, °C		−46 ± 7	
Time to non‐sustained isolation, s		187 ± 70	
Temperature at non‐sustained isolation, °C		−48 ± 4	
Minimum temperature, °C		−52 ± 6	
Total application number, *n*		2.5 ± 1.4	

Abbreviations: CS, coronary sinus vein; LAAI, left atrial appendage isolation; PV, pulmonary vein; PVI, pulmonary vein isolation.

In the CB group, we noted a higher BMI, a greater number of male patients, a higher prevalence of coronary artery disease, and a higher number of previous AF ablation procedures.

**FIGURE 2:**
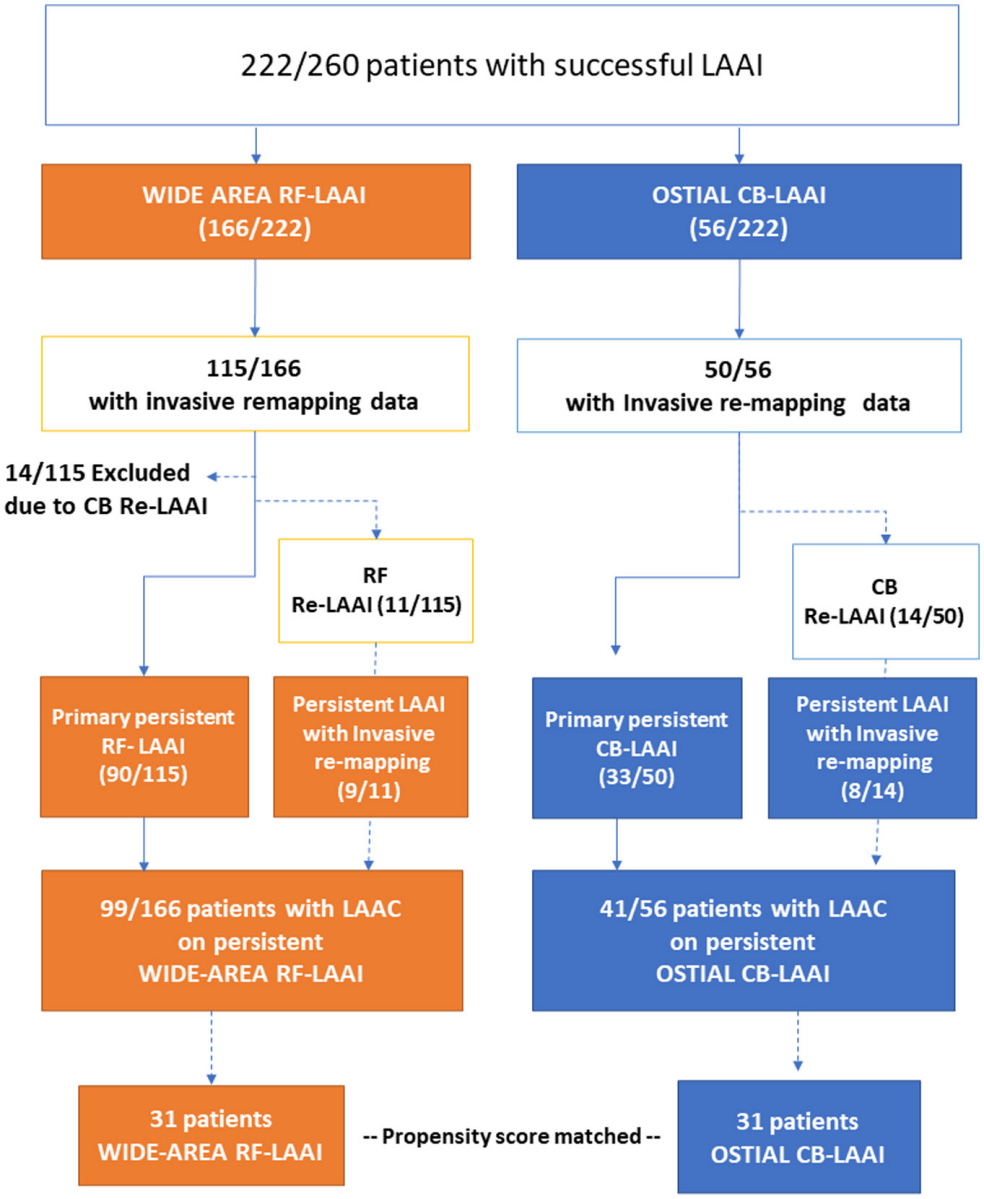
Study flow chart

The detailed study flowchart is summarized in Figure 2. A follow‐up analysis was conducted on 140 patients with durable LAAI who underwent LAAC (RF: *n* = 99, CB: *n* = 41).

### 3.2 Results of the index LAAI procedure

**FIGURE 3:**
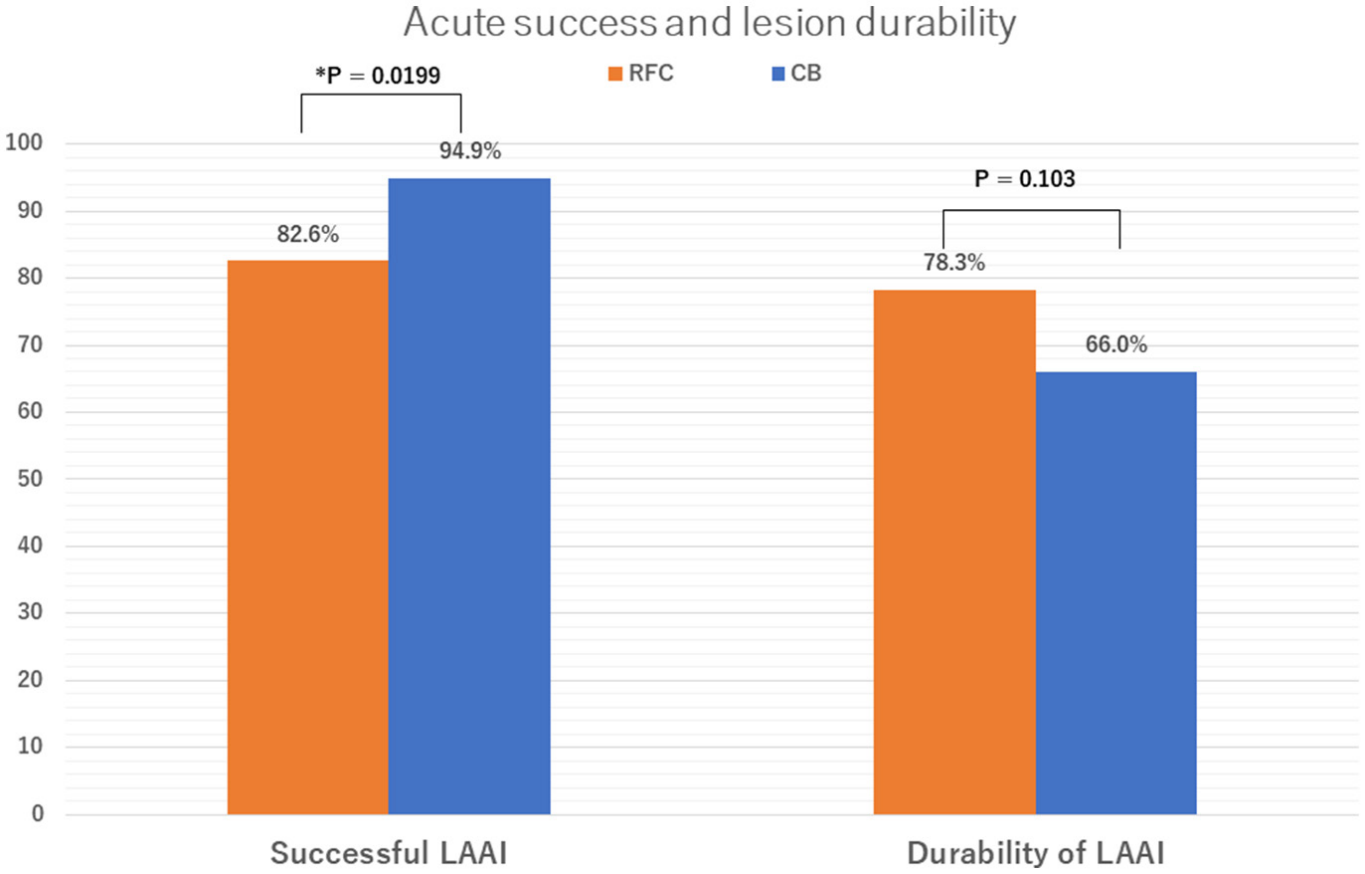
Comparison of acute procedural success and lesion durability between RF‐ and CB‐guided LAAI

The rate of acute LAAI was significantly higher in the CB group compared with the RF group (RF group: 82.6% [*n* = 166/201] vs. CB group: 94.9% [*n* = 56/59], *p* = .0199, Figure 3). The reasons for unsuccessful LAAI in the RF group included cardiac tamponade (*n* = 4), incomplete block of the MIL (*n* = 17), AL (*n* = 9), and both lines (*n* = 5).

### 3.3 LAA remapping

Invasive remapping was performed in 115 patients in the RF group and 50 patients in the CB group. The rate of 6‐week durable LAAI was similar between the two groups (RF: 78.3% [*n* = 90/115] vs. CB: 66.0% [*n* = 33/50], *p* = .103, Figure 3).

### 3.4 Results of the repeat LAAI procedure

A total of 25 patients in the RF group and 14 patients in the CB group underwent re‐LAAI.

In the RF group, the patients underwent an RF‐guided (*n* = 11) or a CB‐guided (*n* = 14) re‐LAAI. The latter 14 patients were excluded from the follow‐up to avoid confounding results.

Successful LAAI was achieved in 9 out of 11 patients at the second RF‐guided LAAI. Durable LAAI was documented in all nine patients at the second invasive remapping study.

All 14 patients in the CB group underwent CB‐guided re‐LAAI. Five patients refused an additional remapping procedure, and durable LAAI was documented in eight patients. In one patient, LA to LAA reconduction was documented after two CB‐guided LAAI attempts. Finally, LAAC was performed because of the patients' refusal to undergo a third LAAI attempt (Figure 2).

### 3.5 Procedural safety in LAAI procedure

Procedural complications were recorded in 8.0% and 6.8% of the patients in the RF and CB groups, respectively (*p* = .765). Full details of complications are summarized in Table 3. A total of 15 (7.5%) pericardial effusions were noted after RF‐guided LAAI (vs. 0% in CB‐guided LAAI, *p* = .0262). The rate of cardiac tamponade was numerically higher in the RF than in the CB group (RF: 6.0% (*n* = 12) vs. CB: 0% (*n* = 0), *p* = .074). Along with the learning curve, the rate of cardiac tamponade was reduced in five cases in the first quarter tile (Q1), four in the Q2, two in the Q3, and one in the Q4. In 4 of these 12 cases, the procedure was terminated without achieving LAAI because of procedural complications. Cardiac surgery was not required in any of the included patients.

**TABLE 3:** Summary of complications during the perioperative period and follow‐up

	RF, *N* = 201	CB, *N* = 59	*p*
Total acute procedural complications, %	8.0	6.8	.792
Pericardial effusion, %	7.5	0.0	.0262
Cardiac tamponade, %	6.0	0.0	.074
Pericardial effusion without pericardiocentesis	1.5	0.0	1.000
Phrenic nerve paralysis, %	0	3.4	.051
Cerebral stroke, %	0	0.0	1.000
Puncture site trouble, %	0.5	3.4	.130
Thrombus formation at remapping study, %	11.1	9.8	1.000
Complications during follow‐up, %	RFC, *N* = 99	CB, *N* = 41	
Death, %, *n*	2.0 (2)	2.4 (1)	
Total stroke event, %, *n*	4.0 (4)	0 (0)	
Stroke before LAAC, %, *n*	1.0 (1)	0.0 (0)	
Stroke after LAAC, %, *n*	3.0 (3)	0.0 (0)	
Total bleeding event, %, *n*	2.0 (2)	4.9 (2)	
Intracranial bleeding, %, *n*	1.0 (1)	0.0 (0)	
Gastrointestinal bleeding, %, *n*	1.0 (1)	4.9 (2)	

In the CB group, two cases of transient left phrenic nerve palsy were noted. Access site complications did not differ between groups (RF group: 3.4% vs. CB group 3.4%, *p* = .13).

Despite continued oral anticoagulation, LAA thrombus formation was observed at the invasive remapping study in 11 (11.1%) and 4 (9.8%) patients after RF‐ and CB‐guided LAAI, respectively (*p* = 1.000). No stroke occurred in these patients.

A single stroke event occurred 60 days after LAAI before undergoing LAAC.

### 3.6 Safety during follow‐up

During clinical follow‐up, major bleeding events were documented in two patients in the RF group (one gastrointestinal bleeding and one intracranial bleeding subsequent to stroke) and two patients in the CB group (two gastrointestinal bleeding). The intracranial bleeding subsequent to stroke in the RF group occurred 14 months after LAAC, of which the patient died. Stroke events were recorded in four patients in the RF group. The aforementioned one stroke occurred between LAAI and LAAC under oral anticoagulant therapy with warfarin. The remaining three cases occurred after LAAC. No device leak was observed in these three LAAC procedures. One of three stroke events occurred 1 month after LAAC on dual antiplatelet therapy. A cerebral MRI demonstrated a shower embolic image, which indicated a likelihood of a cardiogenic thromboembolic event. The other two events with a single embolic lesion occurred 12 and 14 months after LAAC. These two patients were on single antiplatelet therapy following the result of absent device‐related thrombus at the control TEE.

Finally, two patients in the RF group (intracranial bleeding or ventricular tachyarrhythmia) and one patient in the CB group (ventricular tachyarrhythmia) died during the study period.

### 3.7 Follow‐up data

**FIGURE 4:**
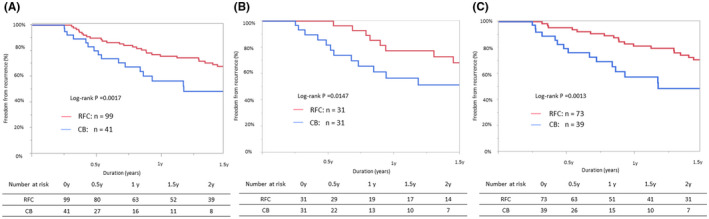
Freedom from atrial tachyarrhythmia (ATa) recurrence after conclusive LAAI procedure in the patients with persistent durable LAAI (a), in the propensity score‐matched cohort (B), and the patients without a history of atrial tachycardia (C)

The median follow‐up period was 28.6 ± 27.2 months. The demographic details of the follow‐up analysis are summarized in Table 1. The 1‐year freedom from ATa recurrence after the last LAAI procedure was significantly higher in the RF than in the CB group (RF: 76.3% vs. CB: 56.7%, *p* = .0017, Figure 4A), as well as in the propensity score matching analysis (RF: 77.5% vs. 56.7%, *p* = .0147, Figure 4B). When patients without atrial tachycardia (RF: *n* = 73, CB: *n* = 39) were analyzed, a higher 1‐year freedom from ATa was also observed in the RF group (RF: 81.3% vs. CB: 57.5%, *p* = .0013, Figure 4C).

The Cox proportional‐hazards regression analysis confirmed RF‐guided LAAI as a predictor of freedom for ATa recurrence (HR: 0.478, 95% confidence interval [CI]: 0.336–0.823, *p* = .017, Table 4).

**TABLE 4:** Predictors for ATa recurrence after the conclusive LAAI procedure

	Unadjusted HR (95% CI)	*p*	Adjusted HR (95% CI)	*p*
Age, years	1.009 (0.981–1.040)	.553	1.010 (0.972–1.037)	.366
Gender, male	1.305 (0.792–2.195)	.299	1.030 (0.556–1.902)	.827
BMI, kg/m^2^	1.003 (0.944–1.061)	.925	–	–
Non‐paroxysmal AF	1.064 (0.603–2.029)	.839	–	–
LA diameter, mm	1.031 (0.983–1.077)	.205	1.039 (0.989–1.082)	.173
Heart failure	1.403 (0.825–2.483)	.216	–	–
RFC‐guided LAAI	0.426 (0.248–0.749)	.0036	0.478 (0.336–0.823)	.017
Coronary artery disease	1.204 (0.640–2.119)	.547	–	–
Number of procedures, *n*	0.780 (0.467–1.204)	.078	0.893 (0.773–1.409)	.336
Previous line ablation	0.850 (0.504–1.404)	.529	0.907 (0.538–1.587)	.578

Abbreviations: CI, confidence interval; HR, hazard ratio.

### 3.8 Predictors of durable LAAI

Our univariate analysis revealed that history of previous AL ablation was the only predictive factor for durable LAAI in RF‐guided LAAI (odds ratio [OR]: 2.98, 95% CI: 1.09–9.60, *p* = .0321). Contrarily, this analysis also revealed that history of previous AL (unadjusted OR: 15.1, CI: 2.60–287.57, *p* = .0010) and RL (unadjusted OR: 6.25, CI: 1.45–43.79, *p* = .0120) ablation was a significant factor for CB‐guided LAAI. Finally, the multivariate analysis revealed that history of previous AL ablation was the only predictive factor for durable LAAI (adjusted OR: 11.85, CI: 1.21–310.24, *p* = .0327).

## 4 DISCUSSION

The main findings of this study are as follows: (1) Higher rate of acute LAAI was achieved by CB ablation. (2) A considerable rate of pericardial effusion was noted in the RF‐guided wide‐area LAAI, although the rate of cardiac tamponade, which required interventional treatment, did not significantly differ. (3) Similar durability of persistent LAAI after a single procedure was documented. (4) Freedom from ATa recurrence with durable LAAI was higher after RF ablation.

### 4.1 Acute procedural success

Our research confirms the findings of a previous study reporting that CB‐guided LAAI can be performed with a high procedural success rate and a low risk for cardiac perforation. However, approximately 1/3 of patients must undergo a repeat ablation procedure for resumed LA to LAA conduction. This prompts the question for the optimal dosing strategy. The thicker myocardium at the LAA neck compared with a PV ostium may require longer freeze duration and/or repeated CB applications.

Contrarily, linear substrate modification using irrigated RF was associated with a considerable number of periprocedural complications. Therefore, ostial RF ablation can be considered as a safer approach that does not compromise the acute LAAI rate in the range of 87%^10^–91%.^11^ Furthermore, coronary arterial vasospasm is also an important concern. No routine coronary artery angiography was performed in the absence of ST‐segment changes in our study, a rare but non‐negligible rate (4%) of the left circumferential coronary artery spasm was observed in the previous report.^12^ Considering the anatomic proximity of the left circumferential artery to the LAA orifice, coronary artery injury should be avoided. The feasibility of using the integration of imaging tools to visualize the association of the left coronary arteries with the LAA ostium should be investigated in addition to meticulous observations of ischemic clinical signs.^13^

### 4.2 Durability of LAAI

CB was originally designed only for PVI. The peculiar issue with CB application is that cryothermal energy is directly delivered from the contacting cardiac endocardium tissue and subsequently forms the homogeneous lesion. Thus, this characteristic is exploited to make the superficial lesion on the flat and round tissue. However, LAA ostium is elliptically shaped. Unlike LAA, in which the entire part derives embryonically from the left atrial muscle and remains as remnant, the PV antrum tissue derives embryonically from the primordial pulmonary vein.^14^ This process facilitates the variant regional wall thickness of LAA ostium, which correlates with the frequent acute reconnection area in RF‐guided circumferential LAAI as anterior and superior walls.^14^ As a result of these anatomical differences between LAA and PV, the persistent LAAI durability in this study was lower in CB‐guided LAAI despite the higher acute procedural success rate. However, the higher durability rate was recorded in patients who had already undergone left atrial AL or RL ablation. Theoretically, ostial LAA would be the last structure for an electrical activation front coming through Bachmann's bundle^15^ from the septal side in LA. Blocking this conduction by AL leads to a longer time for LAA^9^ and might contribute to the development of persistent durability. This theory is supported by a previous research study in which the longer time to LAA was found to be the predictor of persistent durable LAAI in CB‐guided procedure (73%).^6^ In patients with a history of anterior linear ablation, CB‐guided ostial isolation resulted in an equivalent persistent durability in LAAI. Another concern is the duration of the application. In our study, a standard application was delivered for 240 s because of safety reason based on a previous report from our group.^9^ The optimal application duration managing the safety and the durability should be investigated with further studies. Lastly, the additional assessment of dormant conduction after LAAI may also be a possible option to improve the long‐term durability.

### 4.3 Clinical follow‐up data

The indication of LAAI in this study was primarily for “PVI non‐responder” patients with an additional electrophysiological diagnosis of LAA. Our data are the first to report and demonstrate the clinical impact of achieving persistent durable LAAI for non‐PV responders using two different techniques. Excluding the report of empirical LAAI at the initial procedure, data on clinical follow‐up are limited. Some studies without proof of durable LAAI described 1‐year ATa‐free survival after RF‐guided wide‐area LAAI in a range of 65%–68.9%.^5^, ^7^ Our group also reported a 75% 1‐year ATa‐free survival following LAAI using two different methods, a finding that is consistent with that of a previous publication.^16^ Since we described the initial experience with both techniques,^5^, ^6^ the indication of CB‐guided LAAI was then expanded for more complicated cases, such as patients with obesity, gender difference, history of coronary artery disease, and multiple procedures refractory, owing to its safety with an increased number of procedures. As a result, the different patient backgrounds may lead to additional possible reasons for the lower freedom from ATa recurrence. Altogether, the better recurrence‐free rate of RF‐guided wide‐area LAAI in this study, compared with CB‐guided ostial LAAI as a sole trigger eliminator, indicated a large amount of tissue involving potential arrhythmogenic substrate in the specific cohort. Additionally, the large area arrhythmogenic substrate modification could be overlapped with potential non‐PV trigger elimination.^17^, ^18^ The future task of appropriate patient selection for LAAI may be identified by additional provocative maneuver assessing non‐PV trigger, e.g. adenosine/isoproterenol challenge, or through the evaluation of atrial substrate, the use of the magnetic response imaging technique.^19^ Furthermore, the upcoming ASTRO‐AF study (ClinicalTrials.gov Identifier: NCT04056390), a prospective randomized study regarding RF‐guided substrate modification vs. a combination of CB‐guided LAAI and subsequent LAAC beyond the durable PVI, will further elucidate the answer to this issue.

Safety is a key concern. In the present study, there was no cardiac tamponade and subsequent stroke after LAAI in the CB group. The perioperative risk–benefit management should be carefully taken into account when deciding the subsequent strategy. The second safety key is thrombus formation after LAAI. The risk of CB‐guided LAAI has been reported as 0%^12^ whereas that of RF‐guided LAAI as 10%–20%.^7^, ^20^ In our study, thrombus formation was recorded in both the RF and CB groups. The durable LAAI was documented in all these cases. For such patients, the feasibility studies of subsequent LAAC in the presence of thrombus have already been reported.^21^, ^22^ Moreover, interventional LAAC after LAAI was reported to reduce the thromboembolic events compared with medical therapy with OAC.^16^ Thus, subsequent LAAC should be planned after LAAI. On the contrary, the thromboembolic risk still remains regardless of device‐related thrombus formation.^16^, ^23^ The electrical left atrial dysfunction by wide‐area LAAI might be associated with the incidence of stroke after LAAC in this study. Considering the indication of LAAC for patients after LAAI, life‐long OAC could be an option to decrease the thromboembolic risk.

### 4.4 Study limitation

Our study has several limitations. First, the retrospective single‐center observational design could have potentially resulted in different patient characteristics, the number of patients, and follow‐up duration in both groups. Thus, potential overlapped characteristics, such as the history of linear ablation, may also exist. To compensate for the baseline difference, we conducted the propensity score matching analysis that, in turn, led to the same conclusion. This ingenuity could improve the statistical confidence. Another major limitation was that RF‐guided LAAI was favored, especially in patients with atrial tachycardia. However, the analysis on patients without a history of atrial tachycardia resulted in the same conclusion. Second, the time point bias might also have affected several procedural and follow‐up results. We embarked on CB‐guided LAAI on the basis of the initial result of RF‐guided LAAI. Therefore, the sample size in the CB group was smaller, and this could statistically underpower the safety item results. Third, the number of patients who underwent the remapping study was smaller in the RF than in the CB group. This is also because of the time point bias. At the early phase of enrollment in this study, patients primarily refused to undergo an additional LAAC procedure after the LAAI procedure. Moreover, since the first case of thrombus formation following LAAI, the rate of remapping study increased. Finally, we defined the period of persistent durable LAAI as 6 weeks because of the risk of thrombus formation. However, there has been no uniform definition of persistent durable LAAI to date.

## 5 CONCLUSION

Procedural left atrial appendage isolation (LAAI) can be more readily and safely achieved by cryoballoon (CB) ablation. Both radiofrequency (RF) and CB techniques showed similar durability of LAAI. RF‐guided wide‐area linear LAAI was associated with higher freedom from ATa recurrence at the expense of a higher risk of pericardial effusion.

## DISCLOSURES

The authors declare that they have no financial conflicts of interest. Drs. Schmidt, Chun, and Chen are consultants to the Biosense Webster. Drs. Schmidt and Chun are consultants to Medtronic. No other relevant conflicts of interest to declare.

## CONFLICT OF INTEREST

No financial relationship concerning the study. Drs. Schmidt, Chun, Chen are consultants to the Biosense Webster. Drs. Schmidt, Chun are consultants to Medtronic. No other relevant conflicts of interest to declare.
